# Long‐ and short‐term outcomes in renal allografts with deceased donors: A large recipient and donor genome‐wide association study

**DOI:** 10.1111/ajt.14594

**Published:** 2018-02-01

**Authors:** Maria P. Hernandez‐Fuentes, Christopher Franklin, Irene Rebollo‐Mesa, Jennifer Mollon, Florence Delaney, Esperanza Perucha, Caragh Stapleton, Richard Borrows, Catherine Byrne, Gianpiero Cavalleri, Brendan Clarke, Menna Clatworthy, John Feehally, Susan Fuggle, Sarah A. Gagliano, Sian Griffin, Abdul Hammad, Robert Higgins, Alan Jardine, Mary Keogan, Timothy Leach, Iain MacPhee, Patrick B. Mark, James Marsh, Peter Maxwell, William McKane, Adam McLean, Charles Newstead, Titus Augustine, Paul Phelan, Steve Powis, Peter Rowe, Neil Sheerin, Ellen Solomon, Henry Stephens, Raj Thuraisingham, Richard Trembath, Peter Topham, Robert Vaughan, Steven H. Sacks, Peter Conlon, Gerhard Opelz, Nicole Soranzo, Michael E. Weale, Graham M. Lord, the United Kingdom and Ireland Renal Transplant Consortium (UKIRTC) and the Wellcome Trust Case Control Consortium (WTCCC)‐3

**Affiliations:** ^1^ King's College London MRC Centre for Transplantation London UK; ^2^ Welcome Trust Sanger Institute Human Genetics Cambridge UK; ^3^ NIHR Biomedical Research Centre at Guy's and St Thomas’ NHS Foundation Trust and King's College London London UK; ^4^ Renal Institute of Birmingham Department of Nephrology and Transplantation Birmingham UK; ^5^ Nottingham Renal and Transplant Unit Nottingham University Hospitals NHS Trust Nottingham UK; ^6^ Royal College of Surgeons in Ireland Dublin Ireland; ^7^ Transplant and Cellular Immunology Leeds Teaching Hospitals NHS Trust Leeds UK; ^8^ Department of Medicine University of Cambridge Cambridge UK; ^9^ Leicester General Hospital Leicester UK; ^10^ Transplant Immunology & Immunogenetics Churchill Hospital Oxford UK; ^11^ Center for Statistical Genetics Department of Biostatistics University of Michigan Ann Arbor MI USA; ^12^ Cardiff & Vale University Health Board Cardiff University Cardiff UK; ^13^ The Royal Liverpool and Broadgreen University Hospitals Liverpool UK; ^14^ University Hospitals Coventry and Warwickshire NHS Trust Coventry UK; ^15^ School of Medicine Dentistry and Nursing University of Glasgow Glasgow UK; ^16^ Queen Alexandra Hospital Portsmouth UK; ^17^ St Georges’ Hospital NHS Trust London UK; ^18^ Epsom and St Helier University Hospitals Trust Carshalton UK; ^19^ School of Medicine Dentistry and Biomedical Sciences Queens University Belfast Belfast UK; ^20^ Sheffield Kidney Institute Sheffield Teaching Hospitals NHS Foundation Trust Sheffield UK; ^21^ Kidney and Transplant Imperial College Healthcare NHS Trust London UK; ^22^ Leeds Teaching Hospitals NHS Trust Leeds UK; ^23^ Central Manchester University Hospitals NHS Trust Manchester UK; ^24^ NHS Lothian Edinburgh UK; ^25^ Division of Medicine University College London London UK; ^26^ Plymouth Hospitals NHS Trust Plymouth UK; ^27^ The Medical School Newcastle University Newcastle Newcastle upon Tyne UK; ^28^ Division of Genetics& Molecular Medicine King's College London London UK; ^29^ Barts Health NHS Trust London UK; ^30^ Clinical Transplantation Laboratory at Guy's Hospital Guy's and St Thomas’ NHS Trust London UK; ^31^ Beaumont Hospital Dublin Ireland; ^32^ University of Heidelberg Transplantation Immunology Heidelberg Germany; ^33^ Department of Haematology University of Cambridge, Cambridge, UK; ^34^ Present address: UCB Celltech Slough UK; ^35^ Present address: Genomics plc Oxford UK

**Keywords:** basic (laboratory) research/science, genomics, graft survival, kidney transplantation/nephrology, rejection, translational research/science

## Abstract

Improvements in immunosuppression have modified short‐term survival of deceased‐donor allografts, but not their rate of long‐term failure. Mismatches between donor and recipient HLA play an important role in the acute and chronic allogeneic immune response against the graft. Perfect matching at clinically relevant HLA loci does not obviate the need for immunosuppression, suggesting that additional genetic variation plays a critical role in both short‐ and long‐term graft outcomes. By combining patient data and samples from supranational cohorts across the United Kingdom and European Union, we performed the first large‐scale genome‐wide association study analyzing both donor and recipient DNA in 2094 complete renal transplant‐pairs with replication in 5866 complete pairs. We studied deceased‐donor grafts allocated on the basis of preferential HLA matching, which provided some control for HLA genetic effects. No strong donor or recipient genetic effects contributing to long‐ or short‐term allograft survival were found outside the HLA region. We discuss the implications for future research and clinical application.

## Abstract 2

The largest multicenter genome‐wide association study to date on short‐ and long‐term renal transplantation outcomes finds no loci outside the HLA region, highlighting challenges in phenotypic heterogeneity and sample size while also providing a reference dataset for future meta‐analyses.

**Abbreviations**
NIHR: The National Institute for Health Research
UKIRTC: The United Kingdom and Ireland Renal Transplant Consortium
WTCCC3: Wellcome Trust Case Control Consortium 3

## 1 INTRODUCTION

Kidney transplantation is a highly successful treatment for end‐stage renal failure, with significant benefits for recipients both in survival and quality of life. Early outcomes have steadily improved over the last 10 years,^1^ with risk‐adjusted and death‐censored, 1‐year renal graft survival rates of 94% and 97% for deceased and living donor transplants, respectively.^2^ However, both late allograft loss and increased mortality among transplant recipients remain key challenges for the transplant community. There are a wide number of factors that are known to influence long‐term transplant outcome, including donor factors such as age and comorbidity, recipient factors such as comorbidity and response to immunosuppression, as well as allograft ischemic time, the degree of HLA mismatch, and the development of donor‐specific antibodies.^3^, ^4^, ^5^ However, a comprehensive understanding of the pathophysiology of graft failure has remained elusive, with the observed variation in patient outcomes still inadequately explained by our current understanding of risk factors. An improved understanding of the determinants of transplantation outcome would allow the development of truly personalized approaches to the management of transplant recipients.

The importance of genetic factors in transplantation has been clear since the inception of the technique, with the first successful kidney transplant having been performed between identical twins in 1954. Renal transplantation between identical twins continues to show excellent long‐term outcomes,^6^, ^7^ and HLA matching has a large impact on graft survival even in the modern era of immunosuppression.^8^

HLA genes are highly polymorphic, and demonstrate the importance of genetic variation in donor‐recipient pairing that impacts on long‐term outcomes. However, over recent decades, our ability to assay human genetic variation beyond the HLA region has increased considerably.

A number of studies have been published over recent years exploring the association between genotypes of interest and renal transplant outcomes.^9^, ^10^ A large proportion of these studies have concentrated on immune‐related genes, based on the hypothesis that the risk of acute rejection or late allograft loss may be modulated by genetic variation in the immune response. As summarized in Table S1, associations have been described between various transplant phenotypes and single nucleotide polymorphisms (SNPs) in a number of genes including those encoding tumor necrosis factor‐α, interleukins‐1, ‐6, and ‐10, and interferon‐γ. Of note, many of these studies have reported inconsistent findings. For example, analysis of DNA from donor‐recipient pairs in the Collaborative Transplant Study failed to replicate an earlier finding that particular combinations of C3 genotypes in the donor and recipient were associated with reduced graft survival.^11^, ^12^ While some of this discrepancy might be explained by methodological or populational differences between these studies, it is difficult to draw firm conclusions about the role of these genetic variations.^13^

More recently, attention has also focused on non‐immune‐related genetic risk variants. Donor genetic variation in *CAV1* (caveolin‐1),^14^ *APOL1* (apolipoprotein‐L1),^15^, ^16^ or *ABCB1* (ATP‐binding cassette, subfamily‐B, member‐1, expressed in the kidney) genes 17, 18 has been reported to be associated with increased risk of allograft failure or poorer recipient survival. Recipient genetic variation effects on graft and patient survival have only been reported in 1 cohort for *CAV1*.^14^ In addition to effects of donor genetic variants, it might be expected that recipient genotypes in other pharmacometabolic pathways might also impact on transplant outcomes such as increased risk of acute rejection.^19^

In general, candidate gene studies in renal transplantation have so far failed to provide consistent and reproducible results. Some of the reasons for this may include small sample sizes, variations in genotyping methodology and strategy, and, perhaps most importantly, a lack of consistency in clinical phenotyping.^20^ Genome‐wide association studies (GWAS) have contributed greatly to an increased understanding of complex common conditions such as inflammatory bowel disease, hypertension, type 2 diabetes, and schizophrenia.^21^ A small number of GWAS have been reported in the field of renal transplantation, describing SNPs associated with cardiovascular adverse events in recipients taking calcineurin inhibitor immunosuppression,^22^ 2 SNPs associated with serum creatinine levels at 5 years posttransplant,^23^ and a number of SNPs associated with the development of new‐onset diabetes after transplantation.^24^ Recently, a GWAS using pooled DNA of recipient‐only origin found variation in 2 new loci associated with acute rejection in both univariate and multivariate analysis.^25^ However, these studies were underpowered for discovery of genetic variants with small effect sizes.

The Wellcome Trust Case Control Consortium (http://www.wtccc.org.uk/ccc3) has led the deployment of GWAS in a wide range of conditions. As part of WTCCC‐3, all renal transplant centers in the United Kingdom and Ireland formed the United Kingdom and Ireland Renal Transplant Consortium (UKIRTC; http://www.ukirtc.org). Collaborative initiatives such as these are essential for the collection of adequate sample numbers, for the sharing of expertise, standardization of techniques, and building consensus on accurate phenotyping of clinical data. Through this consortium, 3936 samples comprising 2094 complete donor‐recipient pairs were tested in the GWAS discovery phase, and an additional 5866 complete donor‐recipient pairs in the replication phase, making this the largest GWAS conducted to date in renal transplantation outcomes.

## 2 METHODS

### 2.1 Discovery study participants

The large multicenter United Kingdom and Ireland Renal Transplant Consortium (http://www.UKIRTC.org), coordinated by King's College London in partnership with the WTCCC‐3 and the National Health Service Blood and Transplant database (NHS‐BT), sourced all available good‐quality stored DNA samples and pre‐existing GWAS data from both recipients and their corresponding donors from all renal transplantation centers in the United Kingdom and the Republic of Ireland (listed in Table S2). The study was approved by the Hammersmith and Queen Charlotte's & Chelsea Research Ethics Committee REC No 08/H0707/1, on October 14, 2009. Third‐party anonymized clinical data were provided by NHS‐BT UK. All samples and anonymized data for the replication cohort were provided by the University of Heidelberg.

**Figure 1:**
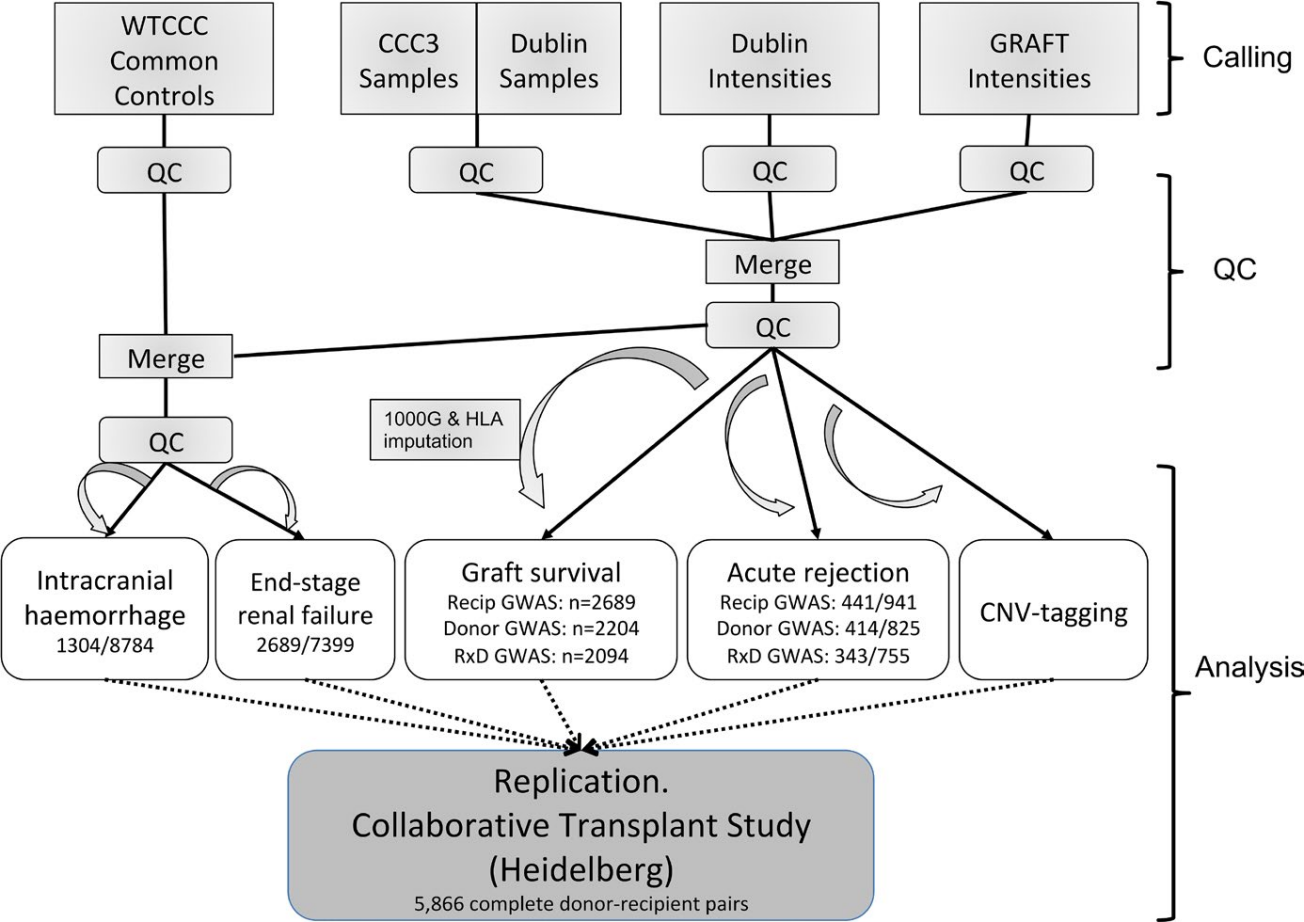
Analysis workflow and strategy. Main input cohorts, analysis methods, and sample sizes are indicated. For binary traits, numbers indicate samples with/without the trait. For further details, see Methods and Supplementary Methods. GWAS, genome‐wide association studies; WTCCC, Wellcome Trust Case Control Consortium

Following genotyping and GWAS quality control (see below), there were 2689 unique recipients, 2204 unique donors, and 2094 complete donor‐recipient transplantation pairs available for analysis. Figure 1 describes the study design and analysis steps. Table 1 provides additional information for the complete donor‐recipient pairs (discrepancies between Figure 1 and Table 1 are because some recipients received more than 1 graft, some donors donated 2 kidneys to different recipients, and not all recipients had matching donor GWAS data and vice versa). The samples and data referred to transplants that took place between December 1981 and December 2007.

**Table 1:** Indicative renal transplant demographics from WTCCC3 and the validation cohort. For consistency, numbers refer to transplants where both donors and recipients passed QC (“complete” donor‐recipient pairs)

	WTCCC3 (post QC)	%	Replication cohort	%
Total transplants where both donors and recipients passed QC	2094	100	5866	100
Total unique donors with a paired recipient	1850	100	5027	100
Total unique recipients with a paired donor	2086	100	5866	100
Mean donor age ± SD	43 ± 15.4		43 ± 16.7	
Mean recipient age ± SD	45 ± 13.3		48 ± 13.6	
0 previous grafts	1864	89	N/A	
1 previous graft	204	9.7	N/A	
2 or more previous grafts	26	1.2	N/A	
Graft survival time (days‐to‐uncensored‐event, 25% quartile)	902		442	
Graft survival time (days‐to‐uncensored‐event, median)	1866		1769	
Graft survival time (days‐to‐uncensored‐event, 75% quartile)	3165		3162	
0 HLA mismatches	223	10.7	N/A	
1 or 2 Class I HLA mismatches	839	40.1	N/A	
1 or 2 Class II HLA mismatches	20	0.96	N/A	
1 or 2 mixed Class I/II HLA mismatches	124	5.9	N/A	
3 to 5 HLA mismatches	612	29.2	N/A	
6 HLA mismatches	8	0.4	N/A	
N/A HLA mismatches	268	12.8	N/A	
Graft survival: total uncensored	495	23.6	2951	50.3
Graft survival: total censored	1599	76.4	2915	49.7
Total double‐kidney transplants	3	0.14	N/A	
Total en bloc kidney transplants	1	0.05	N/A	
Total kidney+pancreas transplants	16	0.76	N/A	
Total kidney‐only transplants	2074	99.0	N/A	
Total rejections (first 3 mo)	259	12.4	N/A	
Total no rejections (first 3 mo)	915	43.7	N/A	
N/A rejections (first 3 mo)	920	43.9	N/A	
Total rejections (3‐12 mo)	221	10.6	575	9.8
Total no rejections (3‐12 mo)	946	45.2	2573	43.9
N/A rejections (3‐12 mo)	927	44.3	2718	46.3

N/A, data not available; QC, quality control; WTCCC3, Wellcome Trust Case Control Consortium‐3.

Inclusion criteria for the study were as follows: (1) deceased donor kidney transplants only; (2) recipient is an adult (>16 years old); (3) reported European ancestry for recipients; and (4) graft survival time >3 months. Donor‐recipient allocation followed NHS‐BT standard UK‐protocols during the study period.

### 2.2 Replication phase participants

A cohort of 5866 complete donor‐recipient pairs, with similar ethnicity to that of the discovery study, were obtained from the Collaborative Transplant Study DNA Biobank held at the University of Heidelberg, Germany (Table 1).

Clinical variables, datasets, and analysis are described in Supplementary Methods.

### 2.3 Discovery phase genotyping and analysis

A whole genome amplification step was undertaken (Source BioScience, Nottingham) for samples containing 5‐40 μL of DNA (n = 990 samples). Samples were assayed via Illumina 670 Quad Custom GWAS chips, and subjected to standard postgenotyping quality control procedures before being imputed to the 1000‐genomes reference dataset. Imputation of HLA alleles from SNP genotype data was undertaken using HLA*IMP software^26^ and compared to serologically typed alleles. A series of GWAS analyses were performed to investigate different genetic models and the renal transplant outcomes of interest. Graft survival genome‐wide analyses were performed (using Cox proportional hazards modeling) for (1) donor SNP genotype main effects; (2) recipient SNP genotype main effects; (3) donor*recipient SNP genotype interaction effects (1df and 3df tests); and (4) CNV‐tag‐SNPs genotype mismatch effects (2 different models). Acute rejection genome‐wide analyses were performed (using logistic regression) for models (1)‐(3). An end‐stage renal failure genome‐wide analysis was performed (using logistic regression) for model (2), and to take advantage of the opportunity, an intracranial hemorrhage genome‐wide analysis was performed (using logistic regression) for model (1).

### 2.4 Replication‐phase genotyping and analysis

Replication DNA samples were received at King's College London and sent to the Wellcome Trust Sanger Institute for replication analysis. A replication panel of 139 SNPs (post–quality control) was tested based on a combination of low *P* value (<10^−6^) from the discovery phase, plus good support of association signals from SNPs in local linkage disequilibrium (LD), or on prior candidature from previous association studies. SNPs were tested according to the same model as motivated their inclusion in the replication panel (for example, if nominated based on a low *P* value for acute rejection in recipients, then that was also the test of interest in the replication analysis). Meta‐analysis of discovery and replication results was carried out using inverse variance meta‐analysis.^27^

For further details see Figure 1 and the Methods section in the Supplementary Material.

### 2.5 Role of the funding source

The funding sources did not participate in the study design, collection, analysis or interpretation of the data, nor did they have a role in writing the report or the decision to submit for publication.

## 3 RESULTS

### 3.1 SNP association analysis

**Figure 2:**
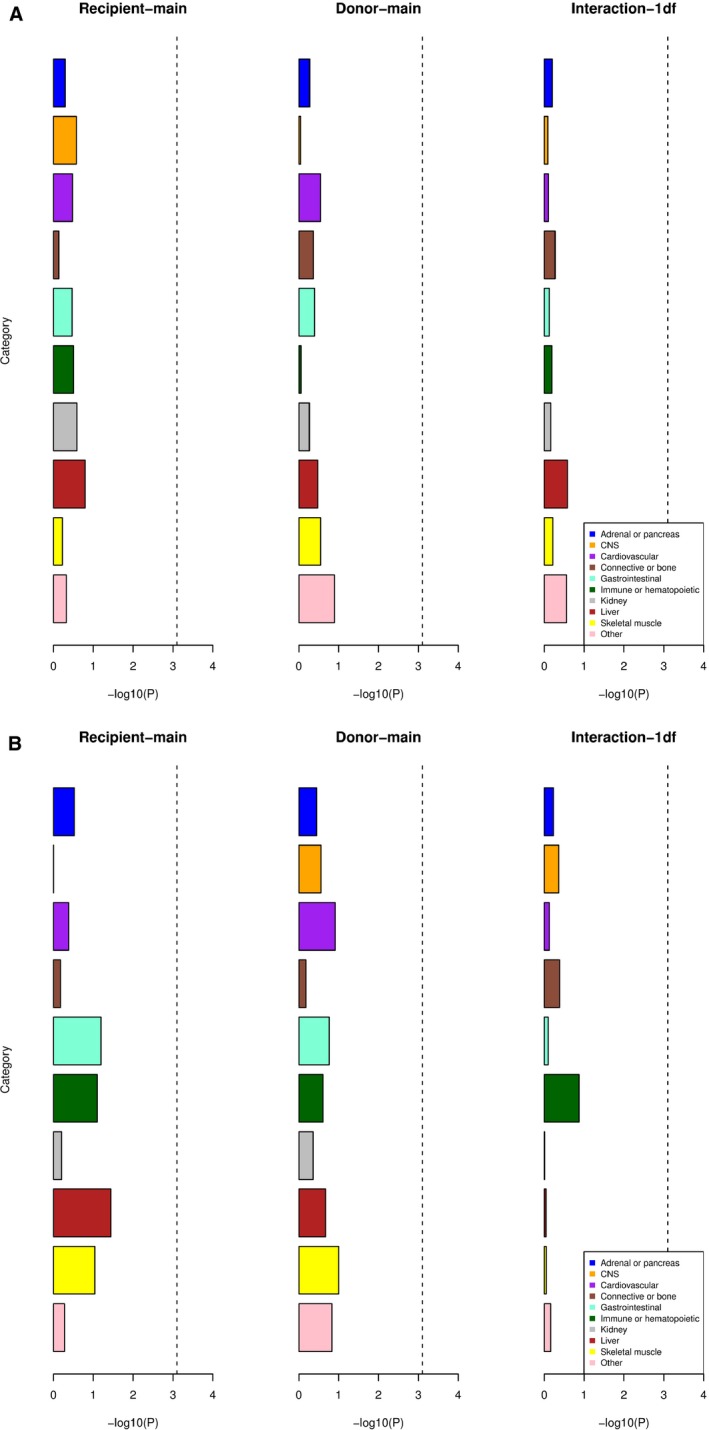
Partitioned heritability analysis of graft survival GWAS results. *X*‐axis indicates –log10 (*P* value) for a test for heritability enrichment within 10 cell/tissue‐type categories of genomic annotations, marking tissue‐ or cell‐type‐specific activity. Dotted lines indicate Bonferroni significance level. (A) Death treated as a censored event; (B) death treated as a failure event. CNS, central nervous system

Despite the large size of our study (Table 1 and Table S2), none of the phenotypes and genetic models tested in the discovery phase produced any LD‐supported single‐SNP results of genome‐wide significance (*P* ≤ 5 × 10^−8^). We also performed a partitioned heritability analysis via stratified LD score regression,^28^ which failed to reveal any significant enrichment of heritability in genomic regions marking tissue‐ or cell‐type‐specific activity (Figure 2).

We pursued a second “replication” phase in the hope that the most significant SNPs would be enriched for true association signals. One hundred thirty‐nine SNPs (post–quality control) were tested, based either on a low *P* value from the discovery phase plus LD support or on prior candidature from previous association studies. In general, the distribution of replication *P* values for all the tests did not depart appreciably from that expected under a global null hypothesis (Table S3). There was some enrichment for low *P* values in the recipient genotype main effect tests for acute rejection in the 12 months following transplantation. However, the *P* values from meta‐analysis (across both discovery and replication phases) did not reach genome‐wide significance (*P* ≤ 5 × 10^−8^). The single SNP with strongest evidence for association was rs2289887 (Figure S1 and Table S4), which had consistent effects in both cohorts but a meta‐analysis *P* value of only 0.00011, indicating that further studies are needed to establish the validity of this signal.

We collated all previously published association signals for early graft rejection and long‐term allograft survival and none of these replicated in our study (Table S1).

### 3.2 HLA mismatch analysis

**Figure 3:**
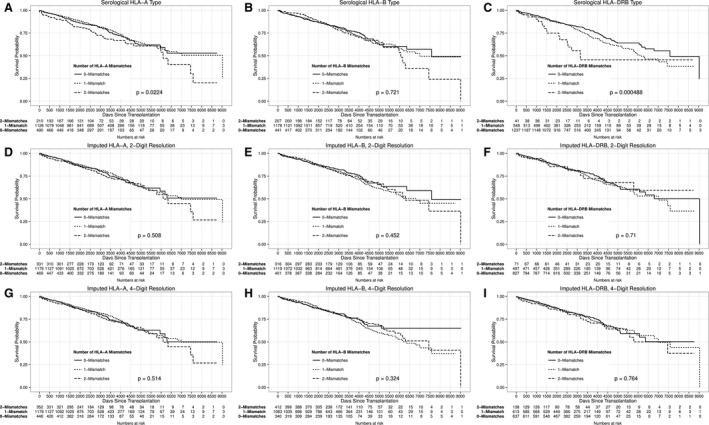
Kaplan‐Meier plots of graft survival by number of mismatches by serological typing (A, B, and C), imputed 2‐digit resolution (D, E, and F), and imputed 4‐digit resolution (G, H, and I). *P* values were obtained from likelihood ratio tests on Cox proportional hazards models

We took advantage of recorded serological information to check for known associations with donor‐recipient mismatch levels and to compare recorded information with imputed mismatches based on SNP genotype information (Figure 3). As expected for deceased donor transplants, the number of non‐zero mismatch transplants in our data was low, reducing our power to detect associations. Nevertheless, we confirmed significant associations with HLA‐A (*P* = .022) and HLA‐DRB (*P* = .00049) mismatches using the recorded data.

On the other hand, the imputed mismatch results did not reproduce the expected associations. We attribute this to 2 factors: (1) the true mismatch = 2 frequency was low, due to donor selection; (2) as with any statistical noise, the imputation inaccuracy tended to act with disproportionate effect in the extremes of the distribution (here, the mismatch = 2 group), resulting in higher relative errors in that group. Thus, although the overall imputation accuracy was high (Tables S5 and S6), the rate of false positives was disproportionately high in the mismatch = 2 categories, swamping our ability to detect the true association signals (Table S7).

## 4 DISCUSSION

In this article, we report the results of the first large‐scale GWAS in renal transplantation. Despite our considerable sample size, we did not replicate any proposed findings from previous candidate gene studies nor did we discover any convincing new variants in our own analyses. There are a number of plausible reasons that may explain this.

Firstly, although this was a study involving thousands of individuals, by GWAS standards it was at the lower end of the range of sample sizes that have been employed for other human traits.^29^, ^30^ A decade of GWAS across multiple complex traits has shown that single effect sizes for any 1 causal variant are typically low, and thus for some traits even bigger sample sizes than ours are needed to discover them. For example, the first robustly associated locus for schizophrenia was found in a discovery GWAS cohort of 3322 cases and 3587 controls.^31^ The number of reliably associated signals for schizophrenia has now grown to 108, providing new biological insights into the disease, thanks to a meta‐analysis that involved 40 000 cases and 113 000 controls.^32^

Secondly, part of our study sought to look for recipient‐donor genetic interactions. Interaction effects require even larger sample sizes to be reliably discovered. With a few notable exceptions,^33^ GWAS studies on other traits have been unsuccessful in discovering reliable interaction effects.

Thirdly, and perhaps most importantly, the transplantation outcomes we considered were relatively crude measures obtained retrospectively from national registry data, collected over many years for reasons other than for acting as endpoints in a genome‐wide association study. Our outcomes were therefore heterogeneous, subject to missingness, and subject to environmental factors that likely weakened the genetic signals. Graft survival time is expected to be subject to a range of factors including graft quality, drug regimen, patient compliance, changes in standard‐of‐care over time, between‐center differences, and underlying biological causes of renal dysfunction. Additionally, a substantial proportion of the survival time data were right‐censored (60.5%‐76.4%, depending on model being fitted), reducing the power for this endpoint.

Acute graft rejection is also a heterogeneous phenotype arising from various immune and nonimmune biological causes. Furthermore, this phenotype was derived from a field that in many records was left blank, resulting in a high degree of missingness (56.7%‐58.3%, depending on model being fitted). The high missingness rate reduced the power of our study, and nonrandom missingness would complicate the interpretation of any positive association signals we might have found (although we note the nonrandom missingness bias would need to be the same in both the discovery and replication cohorts for a signal to be replicated).

Batch effects are also a concern. Both the discovery and replication data were obtained from multiple different collection points in different countries over many years, and thus our phenotypes may be subject to batch effects, for example, arising from different treatment protocols over time and space. Genetic data can also be subject to batch effects, for example, arising from differences in sample collection protocols and unknown differences in population structure. We applied both covariate selection and genetically derived principal component axes to try to mitigate such effects. We also note that, as with nonrandom missingness, the batch effect structure would need to be the same in both the discovery and replication cohorts in order for false‐positive association signals to be replicated. Nevertheless, together these extraneous factors may well have increased the noise in our data, and so reduced (and made less detectible) the genetic effects in our data.

HLA imputation from chip‐based genetic data might in time be of value as an adjunct to serological typing. However, our investigations found that the additional noise introduced by imputation uncertainty prevented the imputed data from picking up the well‐known mismatch signals at HLA‐A and HLA‐DRB. This suggests that the accuracy of HLA imputation will need to be improved before it can be used reliably as an alternative to serotyping.

In contrast, our serological data successfully detected the HLA‐A and HLA‐DRB mismatch signals, despite the reduced power to do so resulting from HLA‐matching of deceased donor allografts. This indicates that, despite the various shortcomings of our study, strong HLA signals were detectable in our data. The implication therefore, at least as far as our primary graft survival endpoint is concerned, is that signals outside the HLA region are weaker than those already established in the HLA region. We therefore anticipate that genetic effects outside of the HLA region are more likely to be of value in elucidating biological pathways than in direct clinical prediction.

We explored this last point further via formal power calculations. These indicated that we were well powered to detect any main effect graft survival association signals involving causal SNPs with allelic hazard ratios in the range 1.4‐1.9 (log‐additive risk model, alpha = 5 × 10^−8^, power = 0.8, minor allele frequency>0.05), and to detect main‐effect acute rejection association signals with allelic odds ratios in the range 1.7‐2.9. We emphasize that these effect sizes are applicable to the traits investigated in this study, but that larger effects might be found in future studies under more precise phenotyping.

In summary, while our study was able to replicate known mismatch signals in the HLA region, we failed to find convincing association signals outside of the HLA region. Both phenotype heterogeneity and sample size may have contributed to this result. Looking ahead, we note that the general lessons from GWAS applied to multiple human traits over more than a decade have brought home 3 clear messages.^29^, ^30^ The first is that all complex traits contain a genetic component, and harbor a large number of causal variants throughout the genome. The second is that larger GWAS studies, often obtained via meta‐analysis with previous studies, inevitably succeed in discovering some portion of these causal variants. The third is that when a large enough portion of these variants is discovered, new insights into the biology of the trait are gained. With these points in mind, we look forward to an international consortium (iGeneTrain^34^), which has been formed to share and meta‐analyze genetic and phenotypic data from most major transplant cohorts worldwide. We also look forward to efforts to collect more detailed phenotypes of relevance to transplant failure, which should provide greater genetic resolution. The data provided by our study form a foundation for ongoing efforts seeking to uncover the biology and improve the prospects for renal transplantation outcomes.

## AUTHOR CONTRIBUTIONS

M.P.H. conducted a literature search, did data and sample collection, manuscript preparation and review, and grant provision and supervision. C.F. and I.R.M. conducted a literature search, did data analysis and interpretation, manuscript preparation and review, and grant provision and supervision. J.M. conducted a literature search. F.D. did data and sample collection. E.P. conducted a literature search, did data and sample collection, did data analysis and interpretation, and manuscript preparation and review. C.S. did data analysis and interpretation. R.B., C.B., B.C., M.C., J.F., S.F., S.G., A.H., R.H., A.J., M.K., T.L., I.Mc., P.B.M., J.M., P.M., W.McK., A.McL., C.H., T.A., P.P., S.P., P.R., H.S., R.T., P.T., did data and sample collection and data analysis and interpretation. N.S. and E.S. did grant provision and supervision. R.T. did study design and grant provision and supervision. R.V. and S.S. did study design, data and sample collection, and data analysis and interpretation. G.C. and P.C. conducted a literature search, did the study design, data and sample collection, manuscript preparation and review, and grant provision and supervision. G.O. did data sample and collection. N.S. did manuscript preparation and review and grant provision and supervision. M.W. did study design, data analysis and interpretation, manuscript preparation and review, and grant provision and supervision. G.L. did study design, manuscript preparation and review, and grant provision and supervision.

## DISCLOSURE

The authors of this article have conflicts of interest to disclose as described by the *American Journal of Transplantation*. M.E.W. is an employee of Genomics plc, a company providing genomic analysis services to the pharmaceutical and health care sectors. M.H.F. and I.R.B. are employees of UCB Celltech, a pharmaceutical company. Their involvement in the conduct of this research was solely in their capacity as academics at King's College London.
