# Supplementary material for: Long‐ and short‐term outcomes in renal allografts with deceased donors: A large recipient and donor genome‐wide association study
Source: Am J Transplant. 2018 Feb 1;18(6):1370–9. doi: 10.1111/ajt.14594 (PMC6001640; doi:10.1111/ajt.14594)
Supplement: Supplementary file 1 [file AJT-18-1370-s001.docx]

**Long and short term outcomes in renal cadaveric allografts: a large recipient and donor genome-wide association study**

**Authors**: UKIRTC, WTCCC3

Maria P Hernandez-Fuentes^1^*, Christopher Franklin^2^*, Irene Rebollo-Mesa^1^*, Jennifer Mollon^1,34^, Florence Delaney^1,3^, Esperanza Perucha^1^, Caragh Stapleton^6^, Richard Borrows^4^, Catherine Byrne^5^, Gianpiero Cavalleri^6^, Brendan Clarke^7^, Menna Clatworthy^8^, John Feehally^9^, Susan Fuggle^10^, Sarah A. Gagliano^11^, Sian Griffin^12^, Abdul Hammad^13^, Robert Higgins^14^, Alan Jardine^15^, Mary Keogan^32^, Timothy Leach ^16^, Iain MacPhee^17^, Patrick B. Mark^15^, James Marsh^18^, Peter Maxwell^19^, William McKane^20^, Adam McLean^21^, Charles Newstead^22^, Titus Augustine^23^, Paul Phelan^24^, Steve Powis^25^, Peter Rowe^26^, Neil Sheerin^27^, Ellen Solomon^28^, Henry Stephens^24^, Raj Thuraisingham^29^, Richard Trembath^28^, Peter Topham^30^, Robert Vaughan^31^, Steven H. Sacks^1,3^, Peter Conlon^6,32^, Gerhard Opelz^33^, Nicole Soranzo^2,34^, Michael E. Weale^28§^, and Graham M. Lord^1,3§^ for the United Kingdom and Ireland Renal Transplant Consortium (UKIRTC) and the Wellcome Trust Case Control Consortium (WTCCC)-3.

* Equal contributors

§ Equal senior contributors

**Corresponding author:** Prof Graham Lord

**email:** [graham.lord@kcl.ac.uk](mailto:graham.lord@kcl.ac.uk)

**Running title:** A GWA study in kidney transplantation

**Abbreviations: (I**nclude Location)

UK: United Kingdom

UKIRTC: The United Kingdom and Ireland Renal Transplant Consortium

WTCCC3: Wellcome Trust Case Control Consortium 3

NIHR: The National Institute for Health Research

# **Supplementary Material**

# **Supplementary Methods**

**Clinical variables**

The following clinical variables were obtained for each sample, either via the centralized National Health Service Blood and Transplant database (for UK centres) or (for selected covariates) from the centre itself (for Beaumont Hospital, Dublin, Ireland): recipient age and sex, donor age and sex, follow-up centre, date of transplant, date of subsequent transplant, date of subsequent registration on transplant list, diabetes as cause of end-stage-renal-failure (yes/no), date of graft failure, cause of graft failure, recipient date of death (if applicable), number of previous grafts, HLA mismatch score, cold ischaemic time, acute graft rejection event 0-3 months post-transplant (yes/no), acute graft rejection event 3-12 months post-transplant (yes/no), donor cause of death by intracranial hemorrhage (yes/no).

From these variables the following primary variables of interest were defined:

1. Graft survival time, defined as days from transplant to graft failure event or censored event, with failure defined as date patient re-registered to the transplant list and patient death treated as a censored event. The graft survival overall percentiles in days were 902 (25%), 1886 (50%) and 3165 (75%).
2. Acute graft rejection (binary variable), defined as any acute rejection event recorded within 12 months post-transplant (derived from the “number of treated rejection episodes” field in NHSBT records). Because acute graft rejection is a high prevalence trait among graft recipients, the comparison group was taken from those recipients who were recorded as not suffering an acute rejection event.
3. End-stage renal failure (binary variable), where all recipients were defined as ‘cases’. Population controls were taken from the WTCCC2 control dataset.
4. Intracranial hemorrhage (binary variable), defined according to donor cause of death. Population controls were taken from the WTCCC2 control dataset.

As a secondary analysis, graft survival time was also defined as above, but with patient death treated as a failure event (on the presumption that a failing graft could contribute to death by other causes). This analysis did not produce any genome-wide significant results, and was not used in selecting SNPs for the replication panel.

As an additional secondary analysis, the acute graft rejection GWAS analyses were reperformed with missing records allocated to the not-suffering group. Missing records were the result of practitioners not filling in the box for “number of treated rejection episodes” in the NHSBT questionnaire. This analysis did not produce any genome-wide significant results, and was not used in selecting SNPs for the replication panel.

For graft survival analyses, a Cox proportional hazards stepwise model selection procedure was applied to the other collected clinical variables (as listed above) to identify the set of the covariates with strongest effect on graft survival, so that these could be accounted for in our genetic association analyses. Stepwise model selection was carried out using the AIC-based ‘step()’ function in R, using standard defaults. The following covariates were identified and used in downstream graft survival GWAS analysis: donor age, recipient age, any previous grafts to recipient (yes/no), regional centre (stratified to three classes). Missing data in covariates is described in Table S8.

**GWAS Genotyping, Quality Control and Imputation (cf. Figure 1 in main text)**

Pre-existing GWAS data, in the form of called genotyped using Illumina’s Genome Studio calling algorithm from Illumina 610-Quad chips, was obtained from King’s College London and Beaumont Hospital.

New samples were quantified using the Quant-iT PicoGreen assay (ThermoFisher) following manufacture’s protocol. Samples which fell between 5 and 40 ng/ul DNA were passed through a whole-genome amplification protocol using the multiple displacement amplification method (™ Source Biosciences, Nottingham) prior to being genotyped on Illumina 610 or 670 or Quad Custom chips and called using the Illuminus genotype calling algorithm.

For end-stage renal failure and intracranial hemorrhage analysis, population control data was obtained from the WTCCC2 National Blood Service and 1958 British Birth Cohort datasets, typed on a combination of Illumina 1M and Affymetrix 6.0 chips.

Initial QC was performed separately on six distinct datasets.

1. WTCCC3 RTD samples (no Whole Genome Amplification).
2. WTCCC3 RTD samples from Whole Genome Amplification.
3. Externally genotyped samples from the Dublin transplant centre.
4. Externally genotyped samples from the KCL GRAFT study.
5. WTCCC2 NBS controls.
6. WTCCC2 1958BC controls.

An initial filter of 95% call rate was applied to SNPs. Sample call rate, heterozygosity and genomic gender was then calculated on the basis of these SNPs. Samples were excluded with call rates below 98%, mean heterozygosity levels +/- 3 standard deviations from the population mean, or if their genomic gender did not match phenotype records.

After exclusion of poor quality samples a subset of SNPs was extracted to perform ethnic ancestry Principal Components Analysis (PCA). These variants had call rate >99%, Minor Allele Frequency (MAF)>5%, Hardy-Weinberg P-value (HWE P)>1x10-4, and were tested for non-random missingness using plink’s--test-mishap option. Pairwise identity-by-decent (IBD) was then calculated and for each pair with genome wide IBD >0.1 (denoting cryptic relatedness) the sample with the lower call rate was excluded.

Each data set was then combined with Hapmap phase3 samples, regions of high LD identified by Price *et al* (1) were excluded, and principal components analysis was performed using EIGENSTRAT. The Hapmap samples were used as the training set and the principal components were projected onto the test samples to identify and exclude samples with clear non-European ancestry.

The six post-QC datasets were then merged and a subset of SNPs that passed QC in all six datasets was extracted. A further IBD check was performed to identify and exclude samples with genetic relationships across studies.

Principal components analysis was performed a second time within the combined cohort to identify axes that correlated with the different study sources. The first four PC axes were used in subsequent association analysis to correct for genetic batch effects, following commonly applied GWAS practice (for technical details see (2) and (3)).

SNP positions in the combined dataset were converted from Build 36 to GRCh37. Individuals were pre-phased using SHAPEITv1 and the 1000-genomes recombination map. The phased data were then imputed to the 1000-genomes Phase 1 integrated variant set (v3) using IMPUTEv2.2.2. Intervals of between 4 and 5 Mb were used with a 250kb buffer and an effective population size of 15,000. SNPs with Info Score >0.4 and MAF>0.01 were used in downstream analyses.

*Imputation of HLA types from genotype data*

With dense SNP genotype data available across the HLA region it is possible to statistically infer the expected HLA class I and II proteins that will be expressed by an individual. In this study, we used the method employed by HLA*IMP (4) which uses a reference set comprised of the 1958 British Birth Cohort ([www.b58cgene.sgul.ac.uk](http://www.b58cgene.sgul.ac.uk/)), CEU samples from the HapMap project (The International HapMap Consortium (5) and additional CEPH CEU samples (6), all of which have dense SNP genotyping and classical HLA types available. For each HLA allele a subset of SNPs present in both the reference data and on our own SNP chip were selected based on their correlation with the classical HLA allele variants. The posterior probability of each possible 4-digit specificity HLA allele variant was calculated based on the SNP genotypes of the samples and the most likely HLA type was determined. Those samples with an assigned posterior probability smaller than 0.9 were considered failed (5.1% of all imputed alleles). Subsequently, the 4-digit-allele variant with the highest posterior probability was assigned. Conversion into 2-digit-types was performed using the conversion tables provided by the WHO Nomenclature Committee for Factors of the HLA System (<http://hla.alleles.org/wmda/rel_dna_ser.txt)>. Similarly, 2-digit types were converted into broad specificities to allow calculation of imputation accuracy at the allele level using serological broad specificity as the reference standard (<http://hla.alleles.org/wmda/rel_ser_ser.txt>).

HLA genotype and serological types reported at the time of transplant were available for both donor and recipient, for at least one locus for 2,094 recipient-donor pairs. Here, we summarise the findings for the A, B and DRB loci only, although all available types were successfully imputed (data not presented). Allele variant call rates were 0.985, 0.967 and 0.891 respectively for A, B and DRB. Call rates were lower than those reported by Dilthey at al. using a posterior probability threshold of 0.70 (4). However, we considered accuracy to be more important than call rate here, due to the need to estimate number of HLA mismatches between donors and recipients. For the number of mismatches to be called, the HLA type of both chromosomes of the donor and recipient had to be successfully imputed. Call rates (against serological data) for the number of HLA mismatches were 0.944, 0.875 and 0.655 for A, B and DRB respectively. The markedly lower call rate for DRB is to be noted, especially given the importance of DRB matching in the context of transplantation.

Imputation accuracy for allele variants was estimated by comparing the derived-imputed broad specificities with broad serological types. Overall accuracy was 98.68%, 97.91% and 98.16% respectively for A, B and DRB (see details by recipient/donor and chromosome in (Table S4).

For the analysis of the impact of serological and imputed number of HLA mismatches between donor and recipient on graft survival, we used univariate Cox-regression models for each locus, and a likelihood ratio test to assess statistical significance.

**Replication study participants and genotyping**

Replication DNA samples were obtained from the Collaborative Transplant Study ( (CTS) DNA Biobank held at the University of Heidelberg, Germany, using the same inclusion criteria as outlined above for the discovery phase (Table1). Following standard DNA quality procedures, 11,218 samples were genotyped at the Wellcome Trust Sanger Institute for 151 SNPs using five Sequenom plexes. After QC procedures 139 SNPs were analyzed on a maximum of 5,866 recipients and 5,027 donors (575 acute graft rejection outcomes, 2573 no acute graft rejection outcomes; 2407 intracranial hemorrhage donors).

Equivalent clinical variables to the discovery study were obtained, and equivalent association tests performed, with the exception that PCA covariates were unavailable so were not fitted, and a country-of-origin covariate was used in place of a regional centre covariate.

**Statistical Analyses**

Since many donor DNA samples were from the same individual (left and right kidney from same deceased donor), and some recipient DNA samples were from the same individual (repeated grafts to same recipient within time period covered by the study), we investigated via simulations the effect on power and type 1 error of using robust (but slow) modelling approaches versus standard modeling approaches which ignored between-sample correlations . We found the differences in power and type 1 error to be modest (data not shown), so for computational efficiency we conducted genome wide analyses using standard modelling procedures. We note that this is only an issue for the donor-recipient interaction tests, since for the donor-only and recipient-only tests we restricted samples to unique donors and unique recipients. Had any significant donor-recipient interaction signals been obtained, we would have sought confirmation via additional robust analyses.

Graft survival was analyzed via Cox proportional hazards modeling. As described above, donor age, recipient age, any previous grafts to recipient (yes/no) and regional centre (stratified to three classes) were fitted as covariates, along with four genetically derived PC axes to account for population structure. For each SNP, the following four tests were carried out as separate GWAS analyses: (1) donor genotype main effect (log-additive genetic model, 1df); (2) recipient genotype main effect (log-additive genetic model, 1df); (3) donor*recipient genotype interaction effect (log-additive genetic models assumed for both donor and recipient genotypes, donor and recipient log-additive main effects fitted as covariates, 1df); and (4) combined donor + recipient + donor*recipient effects (log-additive genetic models assumed for all terms, 3df).

Following the GWAS quality control steps described above, the following sample sizes were obtained for each primary graft survival GWAS:

Graft survival time, patient death treated as censored event, recipient-only genetic model (all unique recipient samples with data entered): n samples = 2689; n failure events = 655.

Graft survival time, patient death treated as censored event, donor-only genetic model (all unique donor samples with data entered): n samples = 2204; n failure events = 544.

Graft survival time, patient death treated as censored event, recipient-donor interaction genetic model (all complete recipient-donor pairs with data entered): n pairs = 2094; n failure events = 495.

Additionally, we performed a secondary analysis in which patient death was treated as a graft failure event:

Graft survival time, patient death treated as failure event, recipient-only genetic model (all unique recipient samples with data entered): n samples = 2512; n failure events = 955.

Graft survival time, patient death treated as failure event, donor-only genetic model (all unique donor samples with data entered): n samples = 2051; n failure events = 810.

Graft survival time, patient death treated as failure event, recipient-donor interaction genetic model (all complete recipient-donor pairs with data entered): n pairs = 1952; n failure events = 751.

Acute graft rejection was analyzed via logistic regression, using transplant outcomes with no acute graft rejection in the first year post-transplant as the comparison group. Four genetically derived PC axes were fitted as covariates. The same set of four models as described above for graft survival were fitted to assess donor, recipient and donor*recipient genotype effects.

Following the GWAS quality control steps described above, the following sample sizes were obtained for each primary acute graft rejection GWAS:

Acute graft rejection (AR), blank records treated as missing (), recipient-only genetic model (all unique recipient samples with data entered): n samples with AR = 441; n samples without AR = 941.

Acute graft rejection (AR), blank records treated as missing, donor-only genetic model (all unique donor samples with data entered): n samples with AR = 414; n samples without AR = 825.

Acute graft rejection (AR), blank records treated as missing, recipient-donor interaction genetic model (all complete recipient-donor pairs with data entered): n samples with AR = 343; n samples without AR = 755.

Additionally, we performed a secondary analysis in which blank records were treated as “no AR event”:

Acute graft rejection (AR), blank records treated as no-AR, recipient-only genetic model (all unique recipient samples with data entered): n samples with AR = 441; n samples without AR = 2254.

Acute graft rejection (AR), blank records treated as no-AR, donor-only genetic model (all unique donor samples with data entered): n samples with AR = 414; n samples without AR = 1904.

Acute graft rejection (AR), blank records treated as no-AR, recipient-donor interaction genetic model (all complete recipient-donor pairs with data entered): n samples with AR = 343; n samples without AR = 1751.

End-stage renal failure was analyzed via logistic regression, using all recipients (not necessarily paired) as ‘cases’ (n=2689) and all donors plus the standard WTCCC2 reference set as ‘controls’ (n=7399). Four genetically derived PC axes were fitted as covariates. Only a recipient main genotype effect was tested (log-additive genetic model assumed), since no plausible causal effect with donor genotype can be expected.

Intracranial hemorrhage was analyzed via logistic regression, using all donors with this as a cause-of-death as ‘cases’ (n=1304) and all other donors plus all recipients plus the standard WTCCC2 reference set as ‘controls’ (n=8784). Four genetically derived PC axes were fitted as covariates. Only a donor main genotype effect was tested (log-additive genetic model assumed), since no plausible causal effect with recipient genotype can be expected.

Copy-number-variant (CNV) mismatch analyses were performed using a set SNP-tags found to tag common CNV variants from the study of Redon *et al* (7). For each tag-SNP, two mismatch variables were defined. misA was set to one if the donor genotype was homozygous for allele A (arbitrarily defined) and the recipient genotype was different, else misA was set to zero; misB was set to one if the donor genotype was homozygous for allele B and the recipient genotype was different, else misB was set to zero. misA and misB were then tested as separate GWAS analyses via Cox proportional hazards modeling on graft survival, using the same clinical and PC covariates as described previously.

Meta-analysis of discovery and replication results was carried out using inverse variance meta-analysis (8).

We applied stratified LD score regression(9) to determine if various categories of genomic annotations (marking tissue- or cell-type-specific activity) were enriched for GWAS heritability. LD score regression exploits the expected relationships between true association signals and local LD around them to correct out systematic biases and arrive at unbiased estimates of genetic heritability within a given set of SNPs (here stratified according to whether they were located within genomic annotation regions). Following Finucane et al.(9), we added annotation categories individually to the baseline model; we used HapMap Project Phase 3 SNPs for the regression and 1000 Genomes Project Phase 3 European population SNPs for the reference panel; we only partitioned the heritability of SNPs with minor allele frequency >5%; and we excluded the MHC region from analysis. The grouped cell-type annotations provided by Finucane et al. are the union of histone marks for 10 broad categories including central nervous system (CNS), cardiovascular, immune/hematopoietic, and liver. For these analyses we corrected for multiple testing of six GWASs across 10 cell-type groups, resulting in a Bonferroni significance threshold of p= 8.3 x 10-4.

**SUPPLEMENTARY BIBLIOGRAPHY**

1. Price AL, Weale ME, Patterson N, Myers SR, Need AC, Shianna KV et al. Long-range LD can confound genome scans in admixed populations. Am J Hum Genet 2008;83(1):132-135; author reply 135-139.

2. Price AL, Patterson NJ, Plenge RM, Weinblatt ME, Shadick NA, Reich D. Principal components analysis corrects for stratification in genome-wide association studies. Nat Genet 2006;38(8):904-909.

3. Patterson N, Price AL, Reich D. Population structure and eigenanalysis. PLoS Genet 2006;2(12):e190.

4. Dilthey AT, Moutsianas L, Leslie S, McVean G. HLA*IMP--an integrated framework for imputing classical HLA alleles from SNP genotypes. Bioinformatics 2011;27(7):968-972.

5. Frazer KA, Ballinger DG, Cox DR, Hinds DA, Stuve LL, Gibbs RA et al. A second generation human haplotype map of over 3.1 million SNPs. Nature 2007;449(7164):851-861.

6. de Bakker PI, McVean G, Sabeti PC, Miretti MM, Green T, Marchini J et al. A high-resolution HLA and SNP haplotype map for disease association studies in the extended human MHC. Nat Genet 2006;38(10):1166-1172.

7. Redon R, Ishikawa S, Fitch KR, Feuk L, Perry GH, Andrews TD et al. Global variation in copy number in the human genome. Nature 2006;444(7118):444-454.

8. Evangelou E, Ioannidis JP. Meta-analysis methods for genome-wide association studies and beyond. Nat Rev Genet 2013;14(6):379-389.

9. Finucane HK, Bulik-Sullivan B, Gusev A, Trynka G, Reshef Y, Loh PR et al. Partitioning heritability by functional annotation using genome-wide association summary statistics. Nat Genet 2015;47(11):1228-1235.

# **Supplementary Results**

**Table S1**: Data on previously published SNPs

(Partial table below for benefit of reviewers. For full table, including explanation of headers, refer to Excel file uploaded in the system)

**Table S2. Sample distribution per Transplant centre. UK**

One row for each renal transplant center. Every centre that performs kidney transplantation in the UK contributed with as many sampes as were locally stored. Please note that the number of donors form each centre is higher as the donors use in the GWA as cadaveric donors could donate more than one kidney to recipients in different centres.

**Table S3. Data on SNPs selected for replication, together with discovery-phase results and replication-phase results.**

(Partial table below for benefit of reviewers. For full table, including explanation of headers, refer to Excel file uploaded in the system)

Yellow cells indicate the discovery-phase test which led to the SNP's inclusion (or an LD proxy of it) in the replication panel.

**Table S4. SNP with evidence for an AR-recipient main effect in both GWAS and Replication.**

| **Details** | **Gene Name ^d^** | **Alternate name** | **Recognised Function** | **Molecular Function HPRD** |
| --- | --- | --- | --- | --- |
| **rs2289887** ^a^  Chrom 8  P_CCC3 = 5.94 e-06  ^b^  ARr.p = 0.0106  ^c^  Meta analysis  p = 0.000115^d^ | *COL14A1* | Collagen, type XIV, alpha 1 | Adhesive role by integrating collagen bundles. The COL2 domain may then interact extracellularly with other matrix molecules or cell surface receptors. | Extracellular matrix structural constituent |
|  | *COLEC10* | collectin sub-family member 10 | Bind to carbohydrate antigens of microorganisms and inhibit infection. They **activate complement through the lectin pathway**, and opsonization by collectin receptors. |  |
|  | *DEPTOR* | DEP domain-containing mTOR-interacting protein | **Negative regulator of the mTORC1 and mTORC2 signaling pathways**. Loss of DEPTOR activates S6K1, Akt, and SGK1, promoting cell growth and survival. |  |
|  | *DSCC1* | Defective in sister chromatid cohesion homolog 1 | Loads PCNA onto primed templates regulating velocity, spacing and restart activity of replication forks. | Protein binding |
|  | ***ENPP2*** | ATX, Autotaxin, | A potent human tumor cell motility-stimulating exophosphodiesterase. Shown to modulate angiogenesis both directly and indirectly.  Lymph Nodes stromal cells constitutively express autotaxin (ATX), an ectoenzyme that is important for the generation of lysophosphatidic acid (LPA). T zone stromal cells **control optimal migratory behaviour of T cells** via multiple signaling cues **mediated by chemokines and ATX/LPA**. | Phosphoric diester hydrolase activity |
|  | *MAL2* | Mal T cell differentiation protein 2 | Member of the machinery of polarized transport. Required for the indirect transcytotic route; from perinuclear endosomes in order for it to travel to the apical surface via a raft-dependent pathway. | Transporter activity |
|  | *MRPL13* | Mitochondrial ribosomal protein L13 | These proteins are implicated in protein synthesis within the mitochondrion. | Structural constituent of ribosome |
|  | *MTBP* | MDM2 Binding Protein | The encoded protein regulates progression through the cell cycle and may be involved in tumour formation, inhibiting cell migration & suppressing invasiveness. | Cell adhesion molecule activity |
|  | *NOV* | IGFBP9, | Immediate-early protein likely to play a role in cell growth regulation. | Growth factor activity |
|  | *TAF2* | TAFII150, Transcription initiation factor TFIID subunit 2 | Stabilizes TFIID binding to core promoter. TFIID is one of the general factors **required for accurate and regulated initiation by RNA polymerase II.** | Transcription factor activity |

a: associated SNP; b: genome-wide p value in the discovery stage; c: p value in the replication stage; d: meta-analysis p value; e: relevant genes within 500 kb of SNP.

**Table S5:** Overall imputation accuracy at the allele level using serological Broad Specificity as gold standard

|  | **A** | **B** | **DRB** |
| --- | --- | --- | --- |
| **Recipient Chr1** | 99.02% | 98.54% | 98.58% |
| **Recipient Chr2** | 98.84% | 97.69% | 98.91% |
| **Donor Chr1** | 98.56% | 97.85% | 97.76% |
| **Donor Chr2** | 98.31% | 97.52% | 97.37% |

Note: Chr1: Chromosome 1, Chr2: Chromosome 2; order being arbitrary

**Table S6.** Frequency distribution of correctly and incorrectly imputed alleles by broad serological specificity

| A | 1 | | 2 | 3 | 9 | 10 | 11 | 19 | 28 |  |  |  |
| --- | --- | --- | --- | --- | --- | --- | --- | --- | --- | --- | --- | --- |
| n(% row) | 1494 (19.2) | | 2259 (29.0) | 1219 (15.7) | 686 (8.8) | 322 (4.1) | 455 (5.8) | 1067 (13.7) | 286 (3.7) |  |  |  |
| INCORRECT (%) | 15 ( 1.0) | | 29 ( 1.3) | 18 ( 1.5) | 10 ( 1.5) | 8 ( 2.5) | 7 ( 1.5) | 12 ( 1.1) | 2 ( 0.7) |  |  |  |
| CORRECT (%) | 1479 (99.0) | | 2219 (98.2) | 1199 (98.4) | 671 (97.8) | 269 (83.5) | 445 (97.8) | 1035 (97.0) | 244 (85.3) |  |  |  |
| NA | 0 ( 0.0) | | 11 ( 0.5) | 2 ( 0.2) | 5 ( 0.7) | 45 (14.0) | 3 ( 0.7) | 20 ( 1.9) | 40 (14.0) |  |  |  |
| B | **5** | | **7** | **8** | **12** | **13** | **14** | **15** | **16** | **17** | **18** | **21** |
| n(% row) | 309 (4.0) | | 1257 (16.1) | 1121 (14.4) | 1501 (19.3) | 131 (1.7) | 239 (3.1) | 520 (6.7) | 216 (2.8) | 317 (4.1) | 287 (3.7) | 143 (1.8) |
| INCORRECT (%) | 7 ( 2.3) | | 22 ( 1.8) | 14 ( 1.2) | 30 ( 2.0) | 1 ( 0.8) | 8 ( 3.3) | 10 ( 1.9) | 8 ( 3.7) | 4 ( 1.3) | 6 ( 2.1) | 5 ( 3.5) |
| CORRECT (%) | 290 (93.9) | | 1219 (97.0) | 1099 (98.0) | 1424 (94.9) | 129 (98.5) | 225 (94.1) | 501 (96.3) | 182 (84.3) | 295 (93.1) | 275 (95.8) | 131 (91.6) |
| NA | 12 ( 3.9) | | 16 ( 1.3) | 8 ( 0.7) | 47 ( 3.1) | 1 ( 0.8) | 6 ( 2.5) | 9 ( 1.7) | 26 (12.0) | 18 ( 5.7) | 6 ( 2.1) | 7 ( 4.9) |
| B (CONTINUED) | **22** | | **27** | **35** | **37** | **40** | **41** | **42** | **47** | **53** | **70** |  |
| n(% row) | 165 (2.1) | | 373 (4.8) | 490 (6.3) | 90 (0.3) | 516 (6.6) | 33 (0.4) | 1 (0.0) | 26 (0.3) | 14 (0.2) | 39 (0.5) |  |
| INCORRECT (%) | 3 ( 1.8) | | 8 ( 2.1) | 11 ( 2.2) | 0 ( 0.0) | 12 ( 2.3) | 1 ( 3.0) | 1 (100.0) | 1 ( 3.8) | 3 (21.4) | 1 ( 2.6) |  |
| CORRECT (%) | 153 (92.7) | | 351 (94.1) | 373 (76.1) | 88 (97.8) | 482 (93.4) | 29 (87.9) | 0 ( 0.0) | 25 (96.2) | 0 ( 0.0) | 30 (76.9) |  |
| NA | 9 ( 5.5) | | 14 ( 3.8) | 106 (21.6) | 2 ( 2.2) | 22 ( 4.3) | 3 ( 9.1) | 0 ( 0.0) | 0 ( 0.0) | 11 (78.6) | 8 (20.5) |  |
| DRB | **1** | **2** | | **3** | **4** | **5** | **6** | **7** | **8** | **9** | **10** | **103** |
| n(% row) | 813 (10.4) | | 1225 (15.7) | 1133 (14.5) | 1657 (21.3) | 567 (7.3) | 858 (11.0) | 1137 (14.6) | 121 (1.6) | 78 (1.0) | 41 (0.5) | 158 (2.0) |
| INCORRECT (%) | 17 ( 2.1) | | 18 ( 1.5) | 18 ( 1.6) | 16 ( 1.0) | 9 ( 1.6) | 15 ( 1.7) | 14 ( 1.2) | 3 ( 2.5) | 3 ( 3.8) | 1 ( 2.4) | 7 ( 4.4) |
| CORRECT (%) | 646 (79.5) | | 1154 (94.2) | 1077 (95.1) | 1260 (76.0) | 326 (57.5) | 742 (86.5) | 1007 (88.6) | 95 (78.5) | 68 (87.2) | 32 (78.0) | 45 (28.5) |
| NA | 150 (18.5) | | 53 ( 4.3) | 38 ( 3.4) | 381 (23.0) | 232 (40.9) | 101 (11.8) | 116 (10.2) | 23 (19.0) | 7 ( 9.0) | 8 (19.5) | 106 (67.1) |

Note: Table includes all available data for the two imputed alleles from all donors and recipients

**Table S7. Predictive performance of imputation compared to serological typing in determining the number of HLA mismatches (at 2-digit resolution) between donor and recipient.**

| **Locus** | **Number**  **Mismatches** | **N**  **Serological** | **N**  **Imputed** | **AUC** | **Sensitivity** | **Specificity** | **PPV** | **NPV** |
| --- | --- | --- | --- | --- | --- | --- | --- | --- |
| **A** | 0 | 479(27.6%) | 424(24.4%) | 0.940 | 0.881 | 0.998 | 0.995 | 0.956 |
|  | 1 | 1057(61.0%) | 1028(59.3%) | 0.920 | 0.921 | 0.919 | 0.946 | 0.881 |
|  | 2 | 196(11.3%) | 280(16.1%) | 0.967 | 0.99 | 0.944 | 0.693 | 0.999 |
| **B** | 0 | 408(25.5%) | 372(23.2%) | 0.949 | 0.902 | 0.997 | 0.989 | 0.967 |
|  | 1 | 1023(63.4%) | 972(60.7%) | 0.926 | 0.915 | 0.938 | 0.963 | 0.862 |
|  | 2 | 171(10.7%) | 258(16.1%) | 0.957 | 0.977 | 0.936 | 0.647 | 0.997 |
| **DRB** | 0 | 852(69.1%) | 744(60.3%) | 0.935 | 0.872 | 0.997 | 0.842 | 0.432 |
|  | 1 | 354(28.7%) | 427(34.6%) | 0.909 | 0.929 | 0.889 | 0.770 | 0.969 |
|  | 2 | 27(2.2%) | 62(5.0%) | 0.967 | 0.963 | 0.970 | 0.419 | 0.999 |

Note: AUC: Area Under the Curve; PPV: Positive Predictive Value; NPV: Negative Predictive Value. Numbers are based on those samples for which serological and genetic data was available. N serological = number of donor-recipient pairs with the number of mismatches in the specific locus as per serological matching. N imputed = number of donor-recipient pairs with the number of mismatches in the specific locus as per imputed HLA definition from donor and recipient.

**Table S8. Missingness in the covariates considered for the multivariate analysis.**

| **Covariate** | **Recorded** | **Missing** |
| --- | --- | --- |
| Recipient Age | 2094 | 0 |
| Donor Age | 2094 | 0 |
| Recipient Gender | 2094 | 0 |
| Donor Gender | 2094 | 0 |
| Followup Centre | 2094 | 0 |
| Transplant Date | 2094 | 0 |
| Diabetes as cause of ESRF | 1981 | 113 |
| Cause of Graft Failure | 1936 * | 158 |
| Patient Cause of Death | 1928 * | 166 |
| Graft Number | 2094 | 0 |
| HLA Mismatches | 1826 | 268 |
| Cold Ischaemia Time | 1409 | 685 |
| Rejection Episodes at <3 Months | 1174 | 920 |
| Rejection Episodes between 3-12 Months | 1167 | 927 |

* Recorded or N/A

**Figure S1.** Regional association plot of the single SNP with main effect tests for acute rejection in the 12 months following transplantation, replication and meta-analysis
